# Sodium/glucose cotransporter 1-dependent metabolic alterations induce tamoxifen resistance in breast cancer by promoting macrophage M2 polarization

**DOI:** 10.1038/s41419-021-03781-x

**Published:** 2021-05-18

**Authors:** Xingjian Niu, Jianli Ma, Jingtong Li, Yucui Gu, Lei Yin, Yiran Wang, Xiaoping Zhou, Jinlu Wang, Hongfei Ji, Qingyuan Zhang

**Affiliations:** 1 Department of Medical Oncology, Harbin Medical University Cancer Hospital, Harbin Medical University, Harbin, 150081 Heilongjiang China; 2 Department of Radiation Oncology, Harbin Medical University Cancer Hospital, Harbin, 150081 Heilongjiang China; 3 Institute of Cancer Prevention and Treatment, Harbin Medical University, Harbin, 150081 Heilongjiang China; 4 Heilongjiang Academy of Medical Sciences, Harbin, 150081 Heilongjiang China

**Keywords:** Breast cancer, Cancer metabolism

## Abstract

Endocrine therapy is the standard treatment for estrogen receptor (ER)-positive breast cancer, but tumors eventually develop resistance. However, endocrine therapy resistance mechanisms mediated through interactions between breast cancer cells and tumor-associated macrophages (TAMs) are still unclear. Here, we characterized sodium/glucose cotransporter 1 (SGLT1) overexpression drives the highly glycolytic phenotype of tamoxifen-resistant breast cancer cells where enhanced lactic acid secretion promotes M2-like TAM polarization via the hypoxia-inducible factor-1α/signal transducer and activator of transcription-3 pathway. In turn, M2-like TAMs activate breast cancer cells through EGFR/PI3K/Akt signaling, providing feedback to upregulate SGLT1 and promote tamoxifen resistance and accelerate tumor growth in vitro and in vivo. Higher expression of SGLT1 and CD163^+^ TAMs was associated with endocrine-resistant ER-positive breast cancers. Our study identifies a novel vicious cycle of metabolic reprogramming, M2-like TAM polarization, and endocrine therapy resistance, which involves SGLT1, proposing SGLT1 as a therapeutic target to overcome endocrine therapy resistance in breast cancer.

## Introduction

Breast cancer is the most prevalent cancer in women worldwide, and >70% of cases are characterized by estrogen receptor (ER) expression. Standard endocrine therapy reduces the risk of recurrence by ~40%^1,2^; however, the majority of patients, including initial responders, will suffer from relapse^2^. During the past two decades, major breakthroughs have been achieved in the elucidation of antiestrogen therapy resistance mechanisms, and progress in the development of targeted drugs holds promise for treating patients with de novo or acquired endocrine resistance^2–6^. Several potential mechanisms are currently regarded as main contributors to drug resistance, including mutations in the *ESR1* gene^3,4^ activation of survival signaling pathways such as PI3K/Akt/mTOR^5^, and aberrant cell-cycle activation caused by cyclin-dependent kinase 4/6^6^. Nevertheless, since clinical response rates remain poor and many patients do not achieve long-term survival^2^, it is important to identify therapeutic targets that overcome or reverse endocrine therapy resistance in ER-positive breast cancer.

The tumor microenvironment (TME) comprises various immune cells and stromal cells that have profound effects on therapeutic efficacy^7^. Tumor-associated macrophages (TAMs) are the most abundant immune cells in the TME, and functional TAM polarization is the key feature in promoting tumor cell proliferation and drug sensitivity^8^. We have previously demonstrated that TAMs in the TME in postmenopausal breast cancer patients are predominantly polarized toward an anti-inflammatory macrophage (M2) phenotype^9^, which is associated with tamoxifen resistance and reduced survival^10^. However, the mechanisms by which TAMs are involved in endocrine therapy resistance in ER-positive breast cancer remain unclear.

The immunosuppressive functions of TAMs are regulated not only by receptor ligands, interleukins, and cytokines but also by tumor-derived metabolites accumulated in the TME^11^. Metabolism is now thought to be a key modulator of innate immune cells, in particular macrophages, and the term “metaflammation” describes the interplay between metabolism and immune cells^12^. Indeed, the extraordinarily high aerobic glycolysis rates of cancer cells^13^ promote the accumulation of tumor-derived metabolites in the TME, allowing metabolic reprogramming to occur in TME cellular components including TAMs^14^.

During aerobic glycolysis, glucose uptake is rate-limiting, which occurs through two main glucose transporter (GLUT) families in humans: the concentration-dependent GLUT family and the Na^+^ gradient-dependent sodium/glucose cotransporter (SGLT) family^15^. The basal glucose uptake capacity of SGLTs is considered higher than the GLUTs, especially at low extracellular glucose concentrations^15,16^. SGLT1 is overexpressed in a variety of tumors^17,18^. Tumor cells reportedly exhibit greater metabolic versatility to meet the requirements of malignant growth and to better adapt to various stresses, including limited nutrient availability^13,19^. In addition, under the pressure of many therapeutic treatments, tumor cells usually undergo metabolic reprogramming, which has been reported as the primary cause of drug resistance^20,21^.

We previously reported that long-term tamoxifen treatment-induced endocrine therapy resistance in most ER-positive breast cancers^9,22^. Here, we used metabolomic profiling to reveal that resistant breast cancer cells are characterized by high glucose dependence with further analysis revealing the integral contribution of SGLT1 upregulation. Furthermore, we show that SGLT1 upregulation and accelerated glycolysis underpins the functional interaction between breast cancer cells and the M2 polarization of TAMs. These experimental findings were corroborated in analyses of ex vivo tissues revealing the correlation between upregulation of SGLT1, TAM infiltration, and endocrine therapy resistance in ER-positive breast cancers.

## Materials and methods

### Patients

A total of 189 patients diagnosed with infiltrating ductal breast carcinoma at the Harbin Medical University Cancer Hospital between January 2006 and December 2010 were enrolled in this study. Post-resection primary tumor samples were pathologically confirmed to be ER and/or progesterone receptor (PR) positive via immunohistochemical (IHC) staining with other standard clinicopathological characteristics recorded as required. All patients were treated according to standard practice guidelines, receiving tamoxifen at 20 mg/day for 5 years after chemotherapy or radiation. Patients with distant metastasis (IV stage) or who received neoadjuvant chemotherapy or endocrine therapy before surgery were excluded. Imagological examination or biopsy was conducted to diagnose disease recurrence or metastasis during follow-up. Patients (*n* = 132) who exhibited disease progression or relapsed during adjuvant endocrine therapy with tamoxifen within the 5-year follow-up (<5 years) were included in the tamoxifen-resistant (Tamo-Re) group. We also enrolled 57 patients who did not suffer from recurrence or metastasis within the 5 years into the tamoxifen-sensitive (Tamo-Se) group. Patients groups were matched according to established prognostic factors: age (<50 vs ≥50), tumor size (<2 cm vs ≥2 cm), tumor stage (I vs II vs III), and nodal status (N0 N1 vs N2 N3). All experiments were approved by the Scientific and Ethical Committee of Harbin Medical University and written informed consent was obtained from each patient. No blinding was done for clinical investigation.

### Cell lines and culture conditions

Human breast cancer cell lines, T47D and MCF7, and the human monocytic cell line, THP-1, were obtained from the American Type Culture Collection (ATCC, Manassas, VA, USA). Established stable Tamo-Re cells, MCF7-TAMR1, were purchased from EMD Millipore (Millipore, Burlington, MA, USA). All cell lines were recently authenticated by short tandem repeat profiling and tested for mycoplasma contamination. MCF7 cells were cultured in Dulbecco’s modified Eagle’s medium (DMEM, 4.5 g/L glucose) containing 5% fetal bovine serum (FBS), 10 μg/mL insulin, 100 U/mL penicillin, and 100 μg/mL streptomycin. T47D cells and RPMI-1640 were cultured in RPMI-1640 medium supplemented with 5 or 10% FBS, respectively. MCF7-TAMR1 cells were maintained in DMEM/F12 without phenol red supplemented with 1% FBS, 6 ng/mL insulin, and 1 μM 4-hydroxytamoxifen (MCE, Monmouth Junction, NJ, USA). All cells were maintained in humidified 95% air and 5% CO_2_ at 37 °C.

### Establishment of Tamo-Re breast cancer cells

Tamo-Re breast cancer cell sublines were established by exposing parental cells to 4-hydroxytamoxifen as previously described^19,20^. Briefly, MCF7 and T47D cells were exposed to 1 and 10 μM 4-hydroxytamoxifen, respectively, for 21 days continuously.

### Differentiation of THP-1 cells

THP-1 monocytes were differentiated into macrophages by exposure to 200 nM phorbol 12-myristate 13-acetate (PMA, P8139; Sigma-Aldrich, St. Louis, MO, USA) for 48 h. Differentiated (Mφ) macrophages (5 × 10^6^ cells/well) were cocultured with MCF7-TAMR1 or MCF7 cells (10^6^ cells/well) in Transwell inserts (0.4 μm membrane pore size, 3450; Corning, NY, USA) for 24 h.

### Cell transfections

Cells (1 × 10^6^) were seeded into 6-well plates and infected with lentivirus expressing SGLT1 short hairpin RNA (shRNA) or negative control shRNA (GeneChem, Shanghai, China). The SGLT1 shRNA target sequences were 5′-TCTTCCGCATCCAGGTCAAT-3′ and 5′-GCCTGATGCTATCAGTCATGC-3′, and the negative control sequence was 5′-GAACAATGTTGACCAGGTGA-3′. Alternatively, lentiviruses containing SGLT1 (GeneChem, Shanghai, China) were used to transduce MCF7 cells with selection using 1 μg/mL kanamycin (MCE, Monmouth Junction, NJ, USA). Gene expression changes were confirmed by Western blot.

### Animal studies

Female Nu/Nu nude mice (5 weeks old, 18–20 g, Charles River) were maintained under pathogen-free conditions throughout and fed with sterilized chow and water. Prior to xenografting, mice were randomly divided into four groups (*n* = 10/group) and subcutaneously implanted with an estradiol pellet (0.72 mg/60 days slow release; Innovative Research, USA). The next day, cancer cells mixed with Mφ macrophages (ratio 1:4) in Matrigel (Becton Dickinson, Billerica, MA, USA) solution were injected subcutaneously, and once tumor volumes reached 500 mm^3^, animals were injected subcutaneously with tamoxifen (10 mg/kg) at 4-day intervals. Tumor growth was monitored and measured every 4 days using precision calipers. Tumor cells were collected for immunofluorescence staining. All procedures and experiments were approved by the Scientific and Ethical Committee of the Institute of Harbin Medical University. No blinding was done for animal studies.

### Metabolic profiling

Cells seeded into 6-well plates (2 × 10^5^ cells/well) were cultured for 24 h before collecting conditioned culture medium sample (200 μL) and extracting in water:methanol:acetonitrile (1:2:7) containing 2-chloro-l-phenylalanine after vortexing for 30 s. After further sonication for 10 min and centrifugation at 13,000 × *g* at 4 °C for 15 min, the supernatant was transferred into a fresh 2-mL liquid chromatography–mass spectrometry glass vial for ultra-high-performance liquid chromatography-quadrupole time-of-flight mass spectrometry (UHPLC-Q-TOF-MS) analysis. Supernatants (100 μL) were used for UHPLC-Q-TOF/MS analysis and 10 μL was used for quality control samples. Supernatant metabolic profiling analysis was performed using a UHPLC system (1290; Agilent Technologies, Palo Alto, CA, USA) with a UPLC BEH Amide column coupled to a Triple TOF 6600 (Q-TOF; AB SCIEX, Foster City, CA, USA).

### Glucose uptake, lactic acid production, and extracellular flux assays

Cells were seeded in 6-well plates at 2 × 10^5^ cells/well, and after 24 h, the medium was replaced with serum-free DMEM. After incubation for 12–16 h, the cells and culture medium were collected from each well.

Glucose uptake was measured by flow cytometry in 500 μL of cell culture suspensions containing 10^6^ cells after the addition of 10 μL of 14.6 μM 2-deoxy-2-[(7-nitro-2,1,3-benzoxadiazol-4-yl)amino]-d-glucose (2-NBDG; Invitrogen, Carlsbad, CA, USA) and incubation at 37 °C for 30 min. Cells were then placed on ice with 4 mL of 1× FACS lysing solution. After centrifuging at 200 × *g* at 4 °C for 5 min, glucose uptake in resuspended cell pellets was immediately analyzed with a BD FACS Accuri C6 instrument (FlowJo, San Jose, CA, USA) at excitation and emission wavelengths of 488 and 530 nm, respectively. The cells were washed once more with ice-cold wash buffer and resuspended in 200 μL of ice-cold phosphate-buffered saline (PBS), and again analyzed in the flow cytometer within 10 min.

Extracellular flux assays (Seahorse Bioscience, Chicopee, MA, USA) were used per the manufacturer’s protocol to determine the extracellular acidification rate (ECAR). For each assay, 5 × 10^5^ cells were plated onto XF microplates coated with Cell-Tak (BD Biosciences, San Jose, CA, USA) and incubated for 4 h. RPMI-1640 medium was replaced with glycolysis base media and ECAR was measured under basal conditions after the addition of 1 μM oligomycin and 100 mM 2-deoxy-d-glucose to assess the maximum glycolytic capacity.

The lactic acid concentrations in the media of cells were measured using a Lactate Assay Kit (BioVision, Milpitas, CA, USA). Conditioned culture media were diluted 1:400 with lactate assay buffer for the colorimetric lactate assay. Then, the absorbance was determined at 570 nm using a SpectraMax M5 microplate reader (Molecular Devices) immediately. Background absorbance was removed. The mean values and the s.e.m. for lactate concentration were calculated for each condition.

### ATP level assessment

For ATP detection, cell availability was analyzed using a CellTiter-Glo™ Assay Kit (Promega, Madison, WI, #G7571) according to the manufacturer’s protocol. Briefly, cells were seeded in 96-well plates at 2 × 10^4^ cells/well and then incubated for 24 h. CellTiter-Glo reagent was added to the wells and incubated at room temperature for 10 min with constant shaking. Luminescence intensity was measured by FluoStar Omega plate reader (BMG Labtech).

### Immunohistochemistry

Expression levels of SGLT1 and CD163 were determined by IHC, as previously described^22,23^. Briefly, sections from Tamo-Re and Tamo-Se breast cancer patients were incubated with anti-SGLT1 antibody (1:200, gifted by Dr. Weihua Zhang) or anti-CD163 antibody (1:200, ab87099, Abcam, Cambridge, MA, USA). Tumor sections were scored semiquantitatively as follows: staining extent was scored as 0 (0–5%), 1 (6–25%), 2 (26–50%), or 3 (>50%), and the staining intensity was scored as 0 (absent), 1 (weak), 2 (moderate), or 3 (strong). Specimens with a total score (intensity score multiplied by intensity score) >2 were regarded as positive; otherwise, the specimens were regarded negative. The staining results were independently assessed by two pathologists.

### Immunofluorescence

Cells were seeded onto glass coverslips in 6-well plates, fixed with 4% formaldehyde in PBS, rinsed with PBS, and permeabilized in 0.5% Triton X-100 for 15 min. Samples were then blocked with 3% bovine serum albumin for 30 min, washed with PBS, and incubated with anti-CD68 antibody (1:200, ab213363; Abcam, Cambridge, MA, USA) or anti-CD163 antibody (1:200, ab87099; Abcam, Cambridge, MA, USA) at 4 °C overnight. Subsequently, samples were washed with PBS and incubated with cyanine 3-conjugated anti-mouse antibody (1:400 dilution; Sigma-Aldrich, St. Louis, MO, USA) at 37 °C in the dark for 1 h. Nuclei were counterstained with 4′,6-diamidino-2-phenylindole. Immunofluorescence staining of cells was visualized using a laser confocal scanning microscope (Nikon, Tokyo, Japan).

### RNA extraction and quantitative reverse transcription-PCR (RT-qPCR)

Total RNA extracted using TRIzol (Invitrogen, Carlsbad, CA, USA) was quantitated and reverse-transcribed into complementary DNA (cDNA) using the PrimeScript RT Reagent Kit (Takara, Shiga, Japan) before qPCR analysis with SYBR Premix Ex Taq II reagents (Takara Bio, Otsu, Japan) with reactions containing 5 ng of cDNA, 200 nM each of forward/reverse primers, and iQ SYBR Green Supermix. The cycling conditions were as follows: an initial step of 95 °C for 2 min, followed by 50 cycles of 95 °C for 5 s, 58 °C for 10 s, and extension at 72 °C for 30 s. β-Actin was used as the internal control. The following primer sequences were used: 5′-TCTTCGATTACATCCAGTCCA-3′ (forward) and 5′-TCTCCTCTTCCTCAGTCATC-3′ (reverse) for SGLT1; 5′-TGACGAAGGTCTGTACCGTGTC-3′ (reverse) for SGLT2; 5′-TTGCAGGCTTCTCCAACTGGAC-3′ (forward) and 5′-CAGAACCAGGAGCACAGTGAAG-3′ (reverse) for GLUT1;

5′-TGCCTTTGGCACTCTCAACCAG-3′ (forward) and 5′-GCCATAGCTCTTCAGACCCAAG-3′ (reverse) for GLUT3; 5′-ACTCACCTCTTCAGAACGAATTG-3′ (forward) and 5′-CCATCTTTGGAAGGTTCAGGTTG-3′ (reverse) for interleukin-6 (IL-6); 5′-GATGTCACCGGAGTTGTGC-3′ (forward) and 5′-TGCAGTGTGTTATCCCTGCT-3′ (reverse) for transforming growth factor-β (TGF-β); 5′-TTGGCTTGAGAGACGTGGAC-3′ (forward) and 5′-GTGCCAGTAGCTGGTGTGAA-3′ (reverse) for arginase 1 (ARG1); 5′-TGGCAGCGAGAAACATTCTTTTAT-3′ (forward) and 5′-CAGCAATACTCCGTAAGACCACAC-3′ (reverse) for vascular endothelial growth factor (vegf); 5′-CTCTTGTTGATGTGCTGCTG-3′ (forward) and 5′-GACCTGTTCTTTGAAGTTGACG-3′ (reverse) for IL-1β; 5′-CTCTACAACATCCTGGAGCAAGTG-3′ (forward) and 5′-ACTATGGAGCACAGCCACATTGA-3′ (reverse) for inducible nitric oxide synthase (iNOS); and 5′-ACAGAGCCTCGCCTTTGCCGAT-3′ (forward) and 5′-CTTGCACATGCCGGAGCCGTT-3′ (reverse) for actin.

### Western blotting

Cell cultures were treated with the indicated agents (hypoxia-inducible factor-1α (HIF-1α) inhibitor, PX-478; signal transducer and activator of transcription-3 (STAT3) inhibitor, S3I-201; PI3K inhibitor, wortmannin; or EGFR inhibitor, AG1478; MCE, Monmouth Junction, NJ, USA) for 48 h before washing with PBS and lysis in RIPA buffer containing protease inhibitors. Lysates were clarified by centrifugation at 15,000 × *g* at 4 °C for 10 min and protein concentrations were determined using the BCA assay (Invitrogen, Carlsbad, CA, USA). Equal protein amounts of cell lysate were separated by sodium dodecyl sulfate–polyacrylamide gel electrophoresis and transferred onto polyvinylidene fluoride membranes before blocking with 5% non-fat dried milk for 1.5 h at room temperature and addition of the following primary antibodies at 4 °C overnight: SGLT1 (1:3000, gifted by Dr. Weihua Zhang), CD163 (1:4000, ab87099), ARG1 (1:1000, ab48586), HIF-1α (1:500, ab51608), STAT3 (1:1000, ab119352), VEGF (1:1000, ab69479), EGFR (1:1000, ab40815), PI3K (1:1000, ab191606), p-Akt (1:500, ab8805), and β-actin (1:3000, ab179467) (all purchased from Abcam, Cambridge, MA, USA, unless otherwise indicated). Membranes were washed with Tris-buffered saline containing Tween-20 (TBST), incubated with species-matched horseradish peroxidase-conjugated secondary antibodies at room temperature for 1 h, and washed again with TBST before the addition of ECL reagent. Immunoblots were visualized using a LAS image analyzer (Proteinsimple, San Jose, CA USA) with band intensities quantified using MultiGauge (Fujifilm Life Science, Tokyo, Japan).

### Cell proliferation assay

The effects of tamoxifen on cell proliferation were assessed using the Cell Counting Kit-8 (CCK-8, Vazyme, Nanjing, China) according to the manufacturer’s instructions. Briefly, cell sublines seeded in 96-well plates at a density of 1 × 10^4^ cells/well were allowed to attach for 24 h before the addition of 5 μM tamoxifen for 24, 48, and 72 h. At the end of the respective incubation times, the cells were incubated in a complete medium containing 10% CCK-8 reagent for 2 h and absorbance was measured at 450 nm in a microplate reader (Mannedorf, Switzerland) with values normalized against 0 h measurements.

### Assessment of apoptosis

Apoptosis was evaluated by cytofluorometry using the Annexin V-FITC Kit (Becton Dickinson, Mountain View, CA, USA). Briefly, following the indicated treatments, cells were centrifuged at 100 × *g* for 5 min, washed twice with cold PBS, and then resuspended in 500 µL binding buffer. The cells were then labeled with Annexin V-FITC (5 µL) and propidium iodide (PI, 5 µL) for 15 min in the dark at room temperature and analyzed using a FACS Calibur flow cytometer (Becton Dickinson, Mountain View, CA, USA).

### Enzyme-linked immunosorbent assays

Culture supernatants were collected after 48 h and IL-6, TGF-β, and EGF concentrations determined using commercial ELISA Kits (USCN Life Science, Houston, TX, USA), according to the manufacturer’s instructions.

### Statistics

Analyses were conducted using SPSS (version 22.0; IBM, Armonk, NY, USA) with Student’s *t* test and one-way analysis of variance (used) for analyzing statistical differences between two groups or more than two groups (with similar variance), respectively. *χ*^2^ and Fisher’s exact tests were employed to evaluate the associations between SGLT1 and CD163 expressions and clinicopathological parameters. *P* < 0.05 was considered statistically significant.

## Results

### SGLT1 overexpression correlates with endocrine therapy resistance in ER-positive breast cancer

We first compared the total extracellular metabolite profile of MCF7 breast cancer cells with the established stable Tamo-Re MCF7-TAMR1 subline using UHPLC-MS. For each metabolite, the difference in relative concentration from conditioned to the fresh medium was calculated and normalized to 1 (Supplementary Table 1). The dataset includes 2422 detectable metabolites with normalized differences ranging from 0.6 to 1. In order to better understand the nature of the observed metabolic changes, the altered metabolites were then mapped to metabolic pathways.

**Fig. 1:**
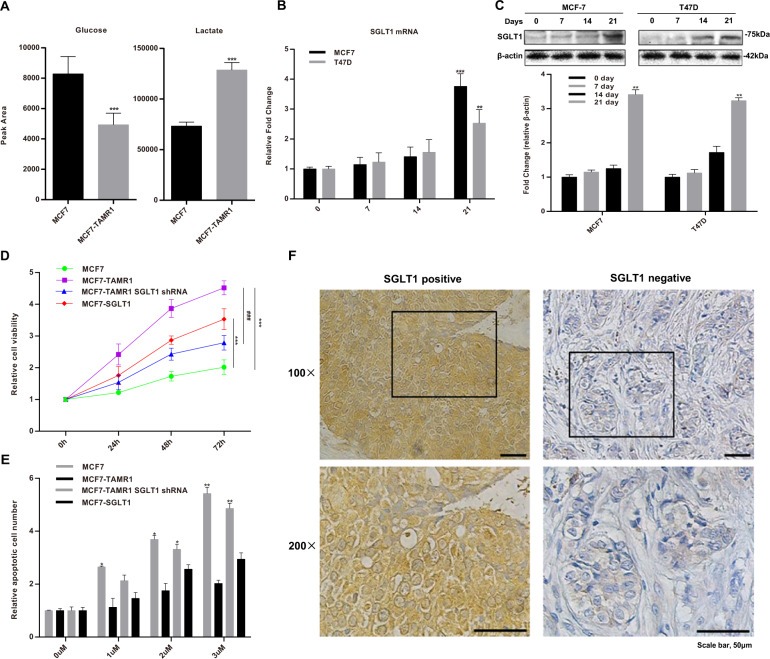
SGLT1 is overexpressed in tamoxifen-resistant ER-positive breast cancer cell lines and tissues. **A** Glucose and lactate levels in 24 h culture supernatants of MCF7 and MCF7-TAMR1 cells determined using UHPLC-MS (***P < 0.001 vs. MCF7). **B**, **C** SGLT1 expression was measured by RT-qPCR (**B**) and Western blot analysis (**C**) after 0, 7, 14, and 21 days of exposure of treatment-naive MCF7 and T47D cells to 1 and 10 μM tamoxifen, respectively. Protein expression changes relative to the actin loading control were determined by densitometry (**P < 0.01, ***P < 0.001 vs. day 0). **D** The cells from MCF7, MCF7-TAMR1, SGLT1 knockdown in MCF7-TAMR1, and SGLT1-overexpressing MCF7 cells were treated with 5 μmol/L tamoxifen and the relative proliferation determined using the CCK-8 assay (***P < 0.001 vs. MCF7; ^###^*P* < 0.001 vs. MCF7-TAMR1). **E** The relative rate of apoptosis was determined in the cells from MCF7, MCF7-TAMR1, SGLT1 knockdown in MCF7-TAMR1, and SGLT1-overexpressing MCF7 cells after treatment with the indicated concentrations of tamoxifen (0–3 μM) for 72 h (*P < 0.05, **P < 0.01 vs. 0 μM). **F** Representative IHC images of SGLT1 expression in Tamo-Re and Tamo-Se tissue samples from patients with ER-positive breast cancer.

Metabolite set enrichment analysis using MetaboAnalyst 3.0 against the significantly altered metabolites between Tamo-Re and control MCF7 cells revealed that the major enrichment in metabolites occurred in pathways involving glycolysis/glucose metabolism (Fig. S1). Indeed, glucose was the most consumed metabolite, significantly decreasing in MCF7-TAMR1 conditioned media compared to parental MCF7 cells (Fig. 1A). In contrast, glutamine levels were hardly affected (Fig. S2A), indicating that the predominant changes in glucose metabolism in Tamo-Re cells occur because of utilizing glucose as the carbon source. Moreover, MCF7-TAMR1 cells exhibited significantly higher lactate and pyruvate production (Fig. 1A and Fig. S2B), consistent with the Warburg effect where glucose is largely converted to pyruvate and lactate. To further supplement these analyses, we integrated the metabolomics data with the Kyoto Encyclopedia of Genes and Genomes pathway analysis, which again pointed to glycolysis/glucose metabolism as being the most significantly altered metabolic pathways associated with tamoxifen resistance (Fig. S3). Besides, the mitochondrial metabolism such as ATP production was significantly increased in MCF7-TAMR1 cells (Fig. S4), suggesting an enhanced metabolic of MCF7-TAMR1 cells.

Extracellular glucose transport into cells represents a key determinant of glucose utilization with GLUTs being overexpressed in many cancer types^15^. To examine whether the altered glucose utilization in Tamo-Re cells was associated with such changes, we analyzed the messenger RNA (mRNA) levels of members of the GLUT and SGLT1 GLUT families, particularly those frequently known to be altered in cancer cells. Instructively, we found that SGLT1 mRNA was increased nearly four-fold in MCF7-TAMR1 vs parental cells, whereas no differences occurred in the expression of SGLT2, GLUT1, and GLUT3 (Fig. S5). These observations propose an important role for SGLT1 in the altered glycolytic phenotype observed in Tamo-Re breast cancer cells.

To further investigate the relationship between SGLT1 and endocrine therapy resistance in ER-positive breast cancer, we investigated the sequential changes in SGLT1 expression that occur during the acquisition of tamoxifen resistance. As used in our prior studies^19,20^ we exposed MCF7 and T47D cells to 4-hydroxytamoxifen for 21 days. Notably, we found that the most obvious change was the increased levels of SGLT1 mRNA at 21 days with pursuant increases in SGLT1 protein levels (Fig. 1B, C). This observation strongly implicates that transcriptional increases in SGLT1 accompany the Tamo-Re phenotype; however, whether this was due to cause or effect remains to be determined.

To verify whether SGLT1 could induce tamoxifen resistance, we also manipulated the levels of SGLT1 by shRNA-mediated knockdown in the MCF7-TAMR1 cells, and, conversely, by overexpressing SGLT1 in parental MCF7 cells (Fig. S6). Thereafter, we assessed the resistance of these to tamoxifen cells along with controls using CCK-8 proliferation assays. As anticipated, cell proliferation rates for MCF7-TAMR1 cells were significantly higher than for MCF7 cells, and, moreover, ectopic expression of SGLT1 increased proliferation above control levels but not to the extent as seen with the MCF7-TAMR1 cells (Fig. 1D). Furthermore, SGLT1 knockdown in MCF7-TAMR1 cells using independent targeting sequences significantly decreases cell proliferation (Fig. 1D), suggesting that SGLT1 levels promote cell proliferation under endocrine blockade. Challenging the cells with higher doses of tamoxifen up to 3 μM indicated that the underlying mechanism for inhibition of proliferation in parental and SGLT1 knockdown in MCF7 cells may rely partly on the induction of apoptosis (Fig. 1E). Together, these data suggest that SGLT1 expression is closely related to endocrine therapy resistance in ER-positive breast cancer.

Finally, it was important to determine if the same changes in SGLT1 were evident in clinically relevant breast cancer samples. Indeed, SGLT1 expression in tumor sections was significantly increased in Tamo-Re patient groups compared to Tamo-Se patient groups (Fig. 1F and Table 1), and was associated with the key clinicopathological characteristics, tumor size, nodal status, and histological grade (Supplementary Table 2). Together, this demonstrates that SGLT1 expression may serve as a marker of tamoxifen resistance in ER-positive breast cancer.

**Table 1:** Difference in SGLT1 expression between Tamo-Re and Tamo-Se groups.

SGLT1	High	Low	*n* (%)	*P* value
Tamo-Re group	72	60	132 (54.5)	0.028
Tamo-Se group	21	36	57 (36.8)	

### SGLT1 overexpression promotes glycolysis in Tamo-Re-resistant breast cancer cells

**Fig. 2:**
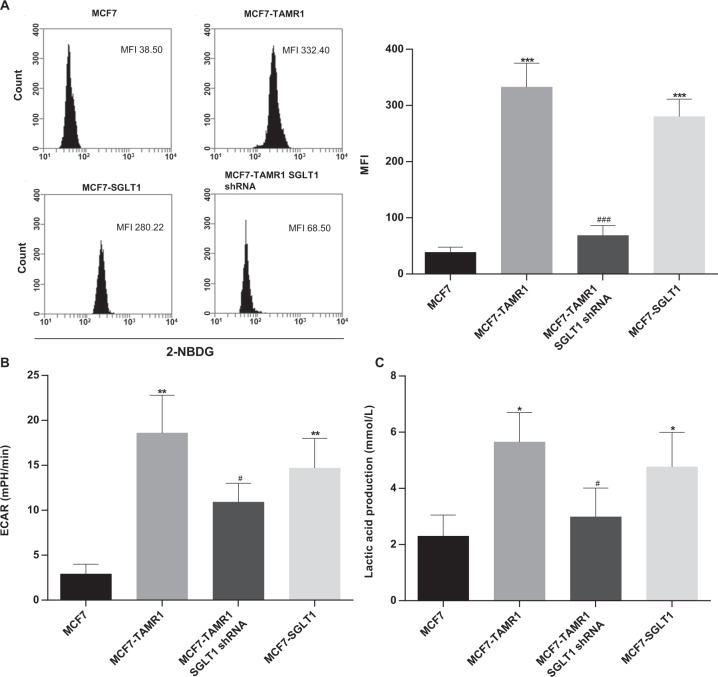
SGLT1 promotes glycolysis in tamoxifen-resistant breast cancer cells. **A** Glucose uptake in MCF7, MCF7-TAMR1, SGLT1 knockdown in MCF7-TAMR1, and SGLT1-overexpressing MCF7 cells as determined by the 2-NBDG assay. MFI median fluorescence intensity (***P < 0.001 vs. MCF7; ^###^*P* < 0.001 vs. MCF7-TAMR1). **B**, **C** ECAR AUC (**B**) and lactic acid production (**C**) in MCF7, MCF7-TAMR1, SGLT1 knockdown in MCF7-TAMR1, and SGLT1-overexpressing MCF7 cells (*P < 0.05, **P < 0.01 vs. MCF7; ^#^*P* < 0.05 vs. MCF7-TAMR1).

We returned to consider the relationship between the highly glycolytic phenotype in tamoxifen resistance breast cancer cells and the function of SGLT1. Using the 2-NBDG method to measure glucose uptake, we first observed that MCF7-TAMR1 cells showed significantly higher glucose uptake than MCF7 cells, and this finding was phenocopied in MCF7 cells overexpressing SGLT1 (Fig. 2A). Finally, the high levels of glucose uptake were reduced to near control levels when SGLT1 expression was silenced in MCF7-TAMR1 cells (Fig. 2A). As expected from the metabolomics data, assessment of ECAR and lactic acid production, proxy measures of glycolysis, also revealed higher levels of ECAR and secreted lactate in the MCF7-TAMR1 subline compared to MCF7 cells (Fig. 2B, C). Moreover, both increased ECAR and lactate production resulted from SGLT1 overexpression, while these measures could also be inhibited, albeit incompletely, by SGLT1 knockdown in MCF7-TAMR1 cells (Fig. 2B, C). These results establish a clear link between the increased glycolytic activity in Tamo-Re breast cancer cells and the expression and function of SGLT1 in promoting glucose uptake.

### SGLT1 overexpression in breast cancer cells promotes M2-like TAM polarization

Functional TAM polarization is known to be regulated by the tumor-derived metabolites^11^, and, conceivably, SGLT1 overexpression may not only impact cancer cells but extend to effects in the TME. To investigate the potential impact of SGLT1 overexpression in breast cancer cells on modulating macrophage differentiation, we employed the human THP-1 monocyte–macrophage differentiation model. Specifically, we tested how coculturing breast cancer cells with macrophages affected their expression of characteristic polarization markers.

**Fig. 3:**
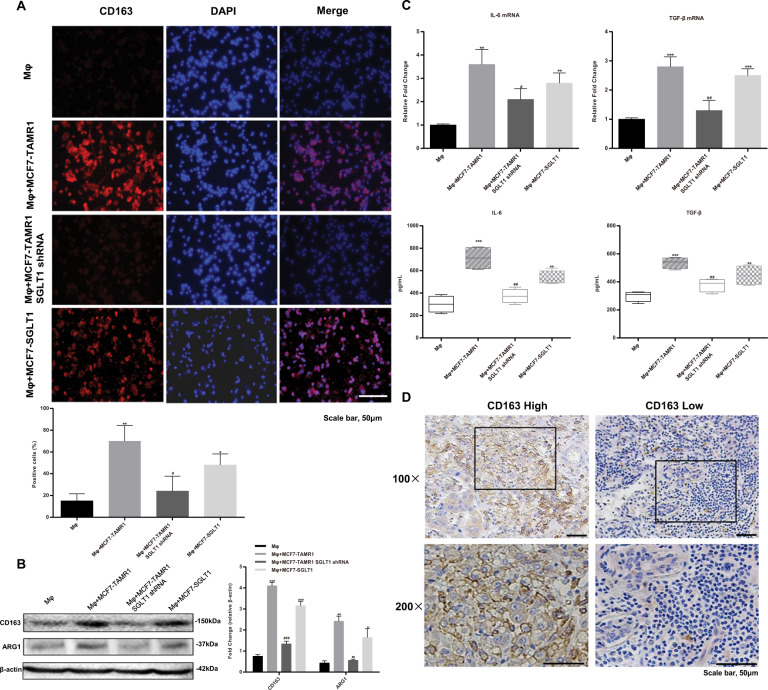
SGLT1-dependent ER-positive breast cancer cells promote M2-like TAM polarization. **A** Representative immunofluorescence staining of CD163 in Mφ macrophages compared with Mφ macrophages cocultured with either MCF7-TAMR1 cells, SGLT1 knockdown in MCF7-TAMR1 cells, or SGLT1-overexpressing MCF7 cells (upper). Quantitation of CD163-positive Mφ macrophages (lower) (*P < 0.05, **P < 0.01 vs. Mφ; ^#^*P* < 0.05 vs. Mφ+MCF7-TAMR1). **B** Western blot analysis of CD163 and ARG1 expression in Mφ macrophages and Mφ macrophage cocultures as per (**A**). Expression changes relative to the actin loading control were determined by densitometry (*P < 0.05, **P < 0.01, ***P < 0.001 vs. Mφ; ^##^*P* < 0.01, ^###^*P* < 0.001 vs. Mφ+MCF7-TAMR1). **C**, **D** mRNA transcript levels of IL-6 and TGF-β measured by RT-qPCR (**C**) or secreted levels of IL-6 and TGF-β measured by ELISA (**D**) in Mφ macrophages and Mφ macrophage cocultures as per (**A**). cocultured with MCF7-TAMR1 cells, with SGLT1 knockdown in MCF7-TAMR1 cells and with SGLT1-overexpressed MCF7 cells (**P < 0.01, ***P < 0.001 vs. Mφ; ^#^*P* < 0.05, ^##^*P* < 0.01 vs. Mφ+MCF7-TAMR1). **E** Representative IHC images of CD163-positive infiltrating cells in Tamo-Re and Tamo-Se tissue samples from patients with ER-positive breast cancer.

Treatment of THP-1 cells for 48 h with PMA results in differentiated Mφ macrophage-like cells displaying an adherent phenotype with extended pseudopods (Fig. S7A), along with the expression of CD68 (Fig. S7B). When THP-1 Mφ macrophages were cocultured with MCF7-TAMR1 cells, there was a notable increase in CD163 expression in the Mφ macrophage (Fig. 3A, B), a cell surface antigen associated with the M2-like phenotype. Instructively, the induced levels of CD163 were significantly inhibited upon shRNA-mediated knockdown of SGLT1 in MCF7-TAMR1 cells and relatively increased in Mφ macrophage cocultured with SGLT1-overexpressed MCF7 cells (Fig. 3A, B). In addition, the expression of ARG1, IL-6, and TGF-β, characteristic markers of M2-like TAMs^24^, was also assessed. Consistent with alterations in CD163, ARG1 expression was higher in Mφ macrophages cocultured with either MCF7-TAMR1 or SGLT1-overexpressing MCF7 cells, and its expression was comparably reduced when SGLT1 was knocked down in MCF7-TAMR1 cells (Fig. 3B). Similarly, cellular mRNA levels of IL-6 and TGF-β together with their extracellular levels were significantly elevated in both Mφ+MCF7-TAMR1 and Mφ+MCF7-SGLT1 cocultures, while again SGLT1 knockdown at least partially reversed these changes (Fig. 3C, D). However, IL-1β and iNOS characteristic markers of M1-like TAMs showed decreased expression in Mφ macrophages cocultured with MCF7-TAMR1 cells (Fig. S8). Thus, from these data, it is evident that SGLT1 overexpression in breast cancer cells can influence macrophage M2 polarization in this experimental setting.

We extrapolated this finding to clinical samples where the levels of infiltrating CD163^+^ TAMs were evaluated by IHC. Comparing the results from the Tamo-Re group after stratifying patients into early tamoxifen resistance (<2 years) vs late developing tamoxifen resistance (2–5 years) cases revealed a greater proportion of cases with higher levels of infiltrating with CD163^+^ TAMs associated with early resistance (Fig. 3E and Table 2). In addition, we found a close positive correlation between CD163^+^ TAMs and SGLT1 expression (Table 3). Taken together with the previous data from the coculture model, these findings propose a causal relationship between high levels of SGLT1 in Tamo-Re breast cancer cells and the M2-like polarization of TAMs.

**Table 2:** Comparison of CD163^+^ macrophage infiltration between early resistance (<2 years) and late resistance (2–5 years) in the Tamo-Re group.

CD163^+^ macrophage	High	Low	*n* (%)	*P* value
Early resistance (<2 years)	63	22	85 (74.1)	0.004
Late resistance (2–5 years)	23	24	47 (48.9)

**Table 3:** CD163^+^ macrophage infiltration was correlated with SGLT1 expression in patients in the Tamo-Re group.

CD163^+^ macrophage	High	Low	*P* value
SGLT1 high	54	18	0.011
SGLT1 low	32	28	

### M2-like TAM polarization is regulated by breast cancer cell-derived lactic acid via the HIF-1α/STAT3 pathway

**Fig. 4:**
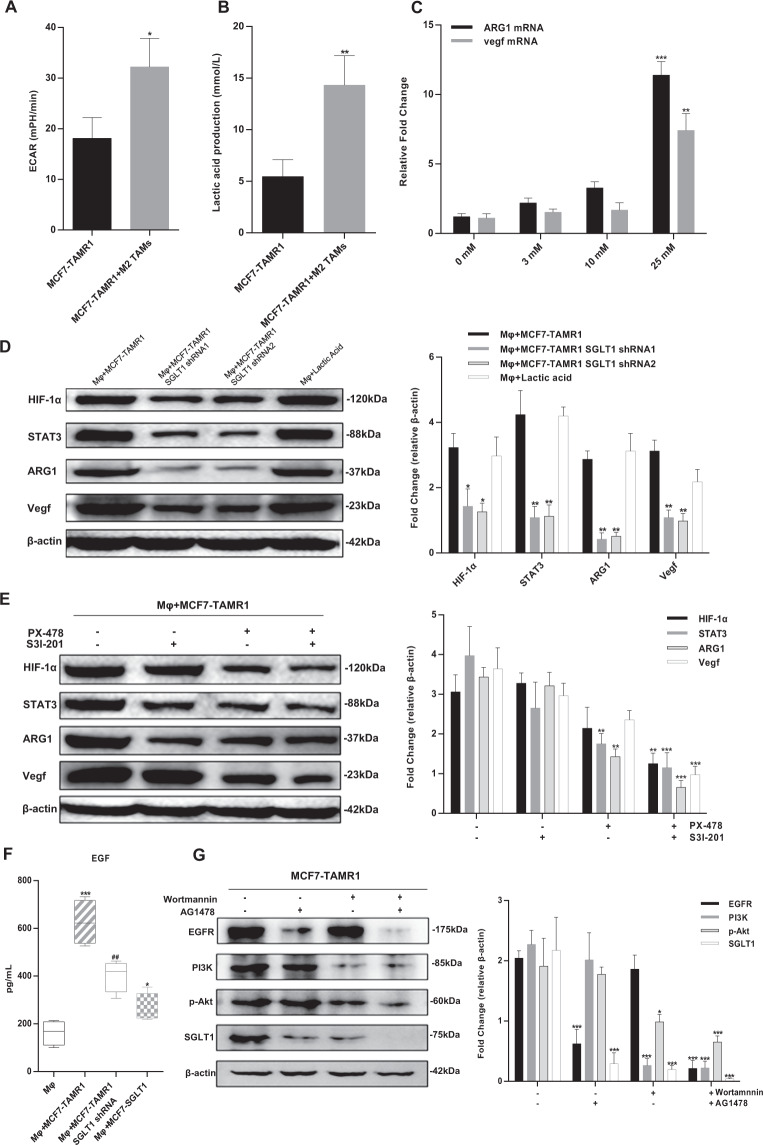
The mechanism of feedback loop of SGLT1 expression in ER-positive breast cancer cells and M2-like TAM polarization. **A**, **B** ECAR AUC (**A**) and lactic acid production (**B**) in MCF7-TAMR1 cells cultured alone or with Mφ macrophages (*P < 0.05, **P < 0.01 vs. MCF7-TAMR1). **C** mRNA transcript levels of ARG1 and vegf mRNA expression in Mφ macrophages treated with 0–25 mM lactic acid (**P < 0.01, ***P < 0.001 vs. 0 mM). **D** Western blot analysis of HIF-1α, STAT3, ARG1, and vegf expression in Mφ macrophages cocultured with MCF7-TAMR1 cells or with SGLT1 knockdown in MCF7-TAMR1 cells. Mφ macrophages treated with 25 mM lactic acid are shown as a control. Expression changes relative to the actin loading control were determined by densitometry (*P < 0.05, **P < 0.01 vs. Mφ+MCF7-TAMR1). **E** Western blot analysis as per (**D**) after treating Mφ macrophages cocultured with MCF7-TAMR1 cells with 25 μM HIF-1α inhibitor PX-478 alone or in combination with 10 μM STAT3 inhibitor S3I-201 (**P < 0.01, ***P < 0.001 vs. negative control). **F** Secreted levels of EGF expression determined by ELISA in culture supernatants from Mφ macrophages or Mφ macrophages cocultured with MCF7-TAMR1 cells, SGLT1 knockdown in MCF7-TAMR1 cells, or with SGLT1-overexpressing MCF7 cells (*P < 0.05, ***P < 0.001 vs. Mφ; ^##^*P* < 0.01 vs. Mφ+MCF7-TAMR1). **G** Western blot analysis of the expression of EGFR, PI3K, p-Akt, and SGLT1 after treating Mφ macrophages cocultured with MCF7-TAMR1 cells with 1 nM PI3K inhibitor wortmannin alone or in combination with 1 μM EGFR inhibitor AG1478 (*P < 0.05, ***P < 0.001 vs. negative control).

To further delineate the mechanisms underlying TAM polarization in Tamo-Re breast cancer, we considered whether the accentuated lactic acid production by cancer cells affects M2-like TAM polarization. We first confirmed the high glycolytic features of the nature of the coculture system where the ECAR and lactic acid secretion of the MCF7-TAMR1 cells were substantially increased when cocultured with Mφ macrophages (Fig. 4A, B). Indeed, lactic acid supplementation alone was sufficient to promote ARG1 and vegf mRNA expression in Mφ macrophages in a dose-dependent manner (Fig. 4C), which increased their cellular protein levels (Fig. 4D). Suggestively, coculture of Mφ macrophages with MCF7-TAMR1 cells also induced high levels of ARG1 and vegf, but the increases were obviated when SGLT1 was knocked down in the MCF7-TAMR1 cells (Fig. 4D). We also conducted parallel measurements of HIF-1α and STAT3 given these are known regulators of ARG1 and vegf expression during M2-like polarization of TAMs^11^. Indeed, there were significantly higher levels of HIF-1α and STAT3 in the Mφ+MCF7-TAMR1 coculture system, which were also reversed by shRNA-mediated knockdown of SGLT1 in MCF7-TAMR1 cells (Fig. 4D). Together, this suggested that the high expression of HIF-1α, STAT3, ARG1, and VEGF in macrophages was facilitated by SGLT1 expression in MCF7-TAMR1 cells.

To verify that ARG1 and vegf expression were regulated by HIF-1α and STAT3 pathways, we assessed the effects of the HIF-1α inhibitor PX-478 and/or the STAT3 inhibitor S3I-201 in the Mφ + MCF7-TAMR1 coculture system. As shown in Fig. 4E, reductions in HIF-1α and STAT3 expression with PX-478 and S3I-201, respectively, confirmed the specific effects of the inhibitors. Moreover, the expression of ARG1 and vegf was inhibited in the presence of PX-478, but not S3I-201, although S3I-201 accentuated the inhibitory effects of PX-478. Integrating these findings with the enhanced lactic acid production through SGLT1 suggests the M2-like polarization of TAMs involves effects mediated through activation of the HIF-1α/STAT3 pathway.

### M2-like TAM feedback regulation of SGLT1 expression in Tamo-Re ER-positive breast cancer cells

Paracrine signaling in the TME is not limited to tumor cell-derived mediators, and M2-like TAMs, in particular, are well known to secrete protumor growth factors^24^. One such factor secreted by TAMs is EGF, which serves to stimulate survival signaling in tumorigenesis^8^. Indeed, secretion of EGF was significantly increased in the Mφ + MCF7-TAMR1 coculture system when compared with the control group, but its levels were suppressed following SGLT1 knockdown in MCF7-TAMR1 cells (Fig. 4F). A significant increase in EGF levels occurred in MCF7 cells overexpressing SGLT1 (Fig. 4F), albeit to a lesser extent.

To investigate the signaling effects of EGF elicited against ER-positive breast cancer cells within the coculture system, we evaluated its effects on the downstream signaling components PI3K and Akt, employing specific inhibitors EGFR kinase activity (AG1478) and PI3K/Akt activation (wortmannin), respectively. Using MCF7-TAMR1 cells as a reference, dual treatment of cells with both inhibitors reduced the expression of EGFR and PI3K and attenuated the activated levels of phospho-Akt (p-Akt Ser 473), as well as downregulated the expression of SGLT1 (Fig. 4G). The total PI3K levels were not impacted by AG1478 treatment alone, but otherwise the findings are consistent with an active EGFR/PI3K/Akt signaling axis in these cells. Of relevance to this report, SGLT1 expression is known to be mediated by several survival signaling pathways^18^, and the suppression of SGLT1 by EGFR and PI3K inhibitors suggests a regulatory role for EGFR/PI3K/Akt signaling in maintaining high levels of SGLT1 expression in these cells.

### The growth-promoting effects of SGLT1 expression in Tamo-Re ER-positive breast cancer cells are recapitulated in vivo

**Fig. 5:**
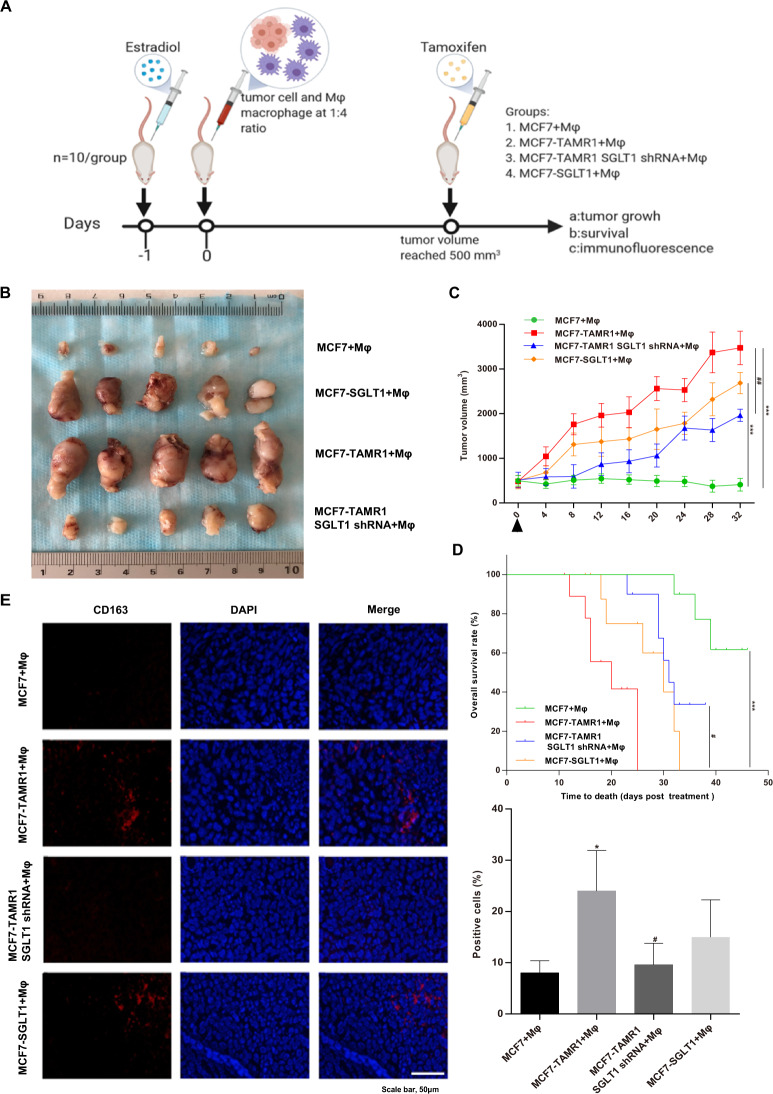
The tumor-promoting effects of the SGLT1-mediated feedback loop in vivo. **A** Illustration of in vivo studies of nude mice injected with cocultures of MCF7/Mφ, MCF7-TAMR1/Mφ, SGLT1 knockdown in MCF7-TAMR1/Mφ, and SGLT1-overexpressing MCF7/Mφ macrophages (1:4 ratio). **B**, **C** Images of dissected xenografted tumors implanted subcutaneously with cocultures of MCF7/Mφ, MCF7-TAMR1/Mφ, SGLT1 knockdown in MCF7-TAMR1/Mφ, and SGLT1-overexpressing MCF7/Mφ after 32 days (**B**). Tumor growth curves determined by caliper measurements shown as mean (mm^3^) ± SEM. *n* = 10/group. **C** After allowing tumors to be established, all mice were treated with 10 mg/kg tamoxifen at 4-day intervals (***P < 0.001 vs. MCF7 + Mφ; ^##^*P* < 0.01 vs. MCF7-TAMR1 + Mφ). **D** Kaplan–Meier plots showing overall survival of mice bearing xenografted tumors implanted subcutaneously with cocultures of MCF7/Mφ, MCF7-TAMR1/Mφ, SGLT1 knockdown in MCF7-TAMR1/Mφ, and SGLT1-overexpressing MCF7/Mφ (***P < 0.001 vs. MCF7+Mφ; ^#^*P* < 0.05 vs. MCF7-TAMR1+Mφ). **E** Representative immunofluorescence staining of CD163 in xenografted tumors implanted subcutaneously with cocultures of MCF7/Mφ, MCF7-TAMR1/Mφ, SGLT1 knockdown in MCF7-TAMR1/Mφ, and SGLT1-overexpressing MCF7/Mφ after 32 days (left). Quantitation of CD163-positive cells (right) (*P < 0.05 vs. MCF7+Mφ; ^#^*P* < 0.05 vs. MCF7-TAMR1+Mφ).

**Fig. 6:**
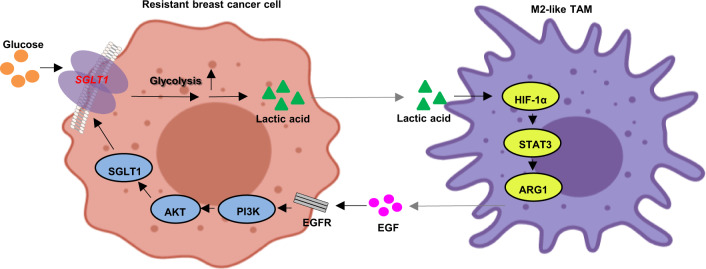
Illustration of the positive feedback loop between tamoxifen-resistant ER-positive breast cancer cells and M2-like TAMs. The glucose transporter SGLT1 is overexpressed in tamoxifen-resistant breast cancer cells, which results in enhanced glycolysis and lactic acid secretion. The breast cancer cell-derived lactic acid promotes M2-like TAM polarization via the HIF-1α/STAT3 pathway. Furthermore, the M2-like TAMs activate the EGFR/PI3K/Akt pathway and ultimately upregulate SGLT1 expression to promote tamoxifen resistance in ER-positive breast cancer cells.

Finally, we sought to validate the tumor-promoting effects of the SGLT1-mediated feedback loop in vivo. Towards this, we compared the growth of subcutaneously implanted xenografts of MCF7, MCF7-TAMR1, SGLT1 knockdown in MCF7-TAMR1, and SGLT1-overexpressing MCF7 cells as Mφ macrophage cocultures since this system mimics interactions between carcinoma cells and effects on TAM polarization (Fig. 5A). After allowing tumors to be established, all mice were treated with tamoxifen to observe effects in the context of tamoxifen resistance (Fig. 5A). Establishing a baseline for these experiments, tumor growth in MCF7/Mφ-injected animals was inhibited in the presence of tamoxifen, while progressive tumor growth occurred in the other experimental groups (Fig. 5B, C). The highest rate of growth was observed in the MCF7-TAMR1/Mφ group followed by SGLT1-overexpressing MCF7/Mφ tumors. Notably, tumor growth in the SGLT1 knockdown in MCF7-TAMR1/Mφ tumors was significantly decreased compared to MCF7-TAMR1/Mφ tumors (Fig. 5B, C). Assessment of survival outcomes in these mice was fully concordant with the growth rates (Fig. 5D). In addition, mice in the MCF7-TAMR1/Mφ group was found a notable increase in CD163 expression, which could be significantly inhibited upon shRNA-mediated knockdown of SGLT1 in MCF7-TAMR1 cells and relatively increased in the SGLT1-overexpressed MCF7/Mφ group (Fig. 5E). Altogether, these data demonstrate the contribution of SGLT1 to tamoxifen resistance in ER-positive breast cancer cells and, moreover, support the importance of the positive feedback loop identified between breast carcinoma cells and M2-like TAMs (Fig. 6).

## Discussion

Endocrine therapy resistance remains a great challenge in the treatment of ER-positive breast cancer. In this study, we report that overexpression of the GLUT SGLT1 in Tamo-Re breast cancer cells substantially accelerates their glycolysis, which in turn impacts M2-like TAM polarization in the TME. Such polarization favors activation of survival signaling in cancer cells towards upregulating SGLT1 expression that promotes tamoxifen resistance in ER-positive breast cancer cells. Thus, our data reveal the existence of a positive feedback loop between metabolic alterations, macrophage polarization, and drug resistance in the TME of ER-positive breast cancer and provide insights towards the development of novel therapeutic strategies for overcoming endocrine therapy resistance.

We observed patent upregulation of SGLT1 in the established MCF7-TAMR1 subline and this phenomenon was recapitulated in two treatment-naive ER-positive breast cancer lines after 3 weeks of exposure to tamoxifen. The impact of high SGLT1 expression resulted in both elevated glucose uptake and utilization as indicated by increased glycolytic flux. The same increase in SGLT1 expression occurred in Tamo-Re breast cancer tissues, indicating that this result was not a culture artifact. Rather, this suggests that Tamo-Re breast cancer tissues may become more dependent on glucose utilization to overcome the effects of long-term exposure to tamoxifen. The question is how this might occur? Probably, the best-known consequence of increased aerobic glycolysis in tumor cells is increased lactic acid efflux and the resulting acidification of the TME. Rather than inhibiting tumor growth, acidosis can positively contribute to tumor development, not the least through effects on immune cells in the TME, which can sense and respond to tumor acidosis^11^.

Macrophages are the most abundant cells within the TME and play pivotal roles in tumor progression by switching from the M1-like antitumor phenotype to the M2-like protumor phenotype^24,25^ M2-like TAMs promote angiogenesis, tumor migration, and invasion, and are also associated with poor prognosis in breast cancer patients^10^. We previously reported high infiltration of M2-like TAMs in Tamo-Re ER-positive breast tumors, which correlated with faster tumor recurrence or metastasis^26^. In this study, we also found a significant increase in M2-like TAM polarization when Mφ cells were cocultured with MCF7-TAMR1 cells. Although it is still not well understood how protumor macrophage polarization is regulated in a Tamo-Re tumor-specific manner, it appears to be central to breast cancer resistance to endocrine therapy.

Lactic acid derived from tumor cells functions as an immunosuppressive mediator, attenuating the differentiation of and cytokine production by T cells and monocytes in vitro and in vivo^11,21,27^. The role of lactic acid in macrophage polarization and function has been recently elucidated. Lactic acid-induced conversion of TAMs to the M2-like phenotype is mainly mediated by HIF-1α, activating STAT-dependent signaling and the anti-inflammatory function of macrophages characterized by the induction of ARG1 and vegf expression^11^. Our results propose that SGLT1 not only facilities a highly glycolytic phenotype, which supports the tamoxifen resistance of breast cancer cells, but also that the metabolic byproduct, lactic acid, participates in the M2-like TAM polarization in the TME, activating the HIF-1α/STAT pathway.

The role of M2-like TAMs in promoting tumor growth and migration has been widely investigated^8,24^. M2-like TAMs release a panel of protumor factors (immunosuppressive factors, growth factors, and cytokines) in the presence of lactic acid to promote tumor progression^28^. Accordingly, we identified that the levels of IL-6 and TGF-β were significantly increased in the medium of Mφ cells cocultured with MCF7-TAMR1 cells, which again could be functionally related to the expression of SGLT1 in the cancer cells. Moreover, we found clear evidence linking the presence of M2-like TAMs in the coculture system with the activation of EGF- and TGF-β-related intracellular signaling pathways, such as the PI3K/Akt pathway in the breast cancer cells. Disruption of the PI3K/Akt pathway in tumor cells inhibits glucose metabolism, suggesting that this pathway might regulate glycolysis^29^. Interestingly, we found that PI3K/Akt signaling was activated upon coculture of M2-like TAMs with MCF7-TAMR1 cells, which further upregulated SGLT1 expression. This process could be reversed by treatment with PI3K and EGFR inhibitors, suggesting that the EGFR/PI3K/Akt axis regulates glycolysis, at least in part, by upregulating SGLT1 expression. In particular, SGLT1 overexpression in tumor cells not only increases glucose uptake but also promotes tumor progression via interaction with the ligand-activated EGFR signaling pathway^30^, suggesting that SGLT1 may have more functions than previously known.

## Conclusions

In conclusion, we propose that a novel vicious cycle exists between metabolic reprogramming, M2-like TAM polarization, and endocrine therapy resistance. Here, our study demonstrates a central role for SGLT1 to allow ER-positive breast cancers to withstand the metabolic stress of endocrine therapy. High SGLT1 expression mediates enhanced glucose uptake and lactic acid secretion, promoting M2-like TAM polarization and feedback activation of EGFR/PI3K/Akt/SGLT1 signaling in tumor cells to enhance tamoxifen resistance. Therefore, targeting SGLT1 may be a potential therapeutic strategy against tamoxifen resistance in breast cancer.

## Supplementary information

Supplement Figure 1

Supplement Figure 2

Supplement Figure 3

Supplement Figure 4

Supplement Figure 5

Supplement Figure 6

Supplement Figure 7

Supplement Figure 8

Table S1

Table S2
