# Supplementary figures and images for: Sodium/glucose cotransporter 1-dependent metabolic alterations induce tamoxifen resistance in breast cancer by promoting macrophage M2 polarization
Source: Cell Death Dis. 2021 May 18;12(6):509. doi: 10.1038/s41419-021-03781-x (PMC8131586; doi:10.1038/s41419-021-03781-x)

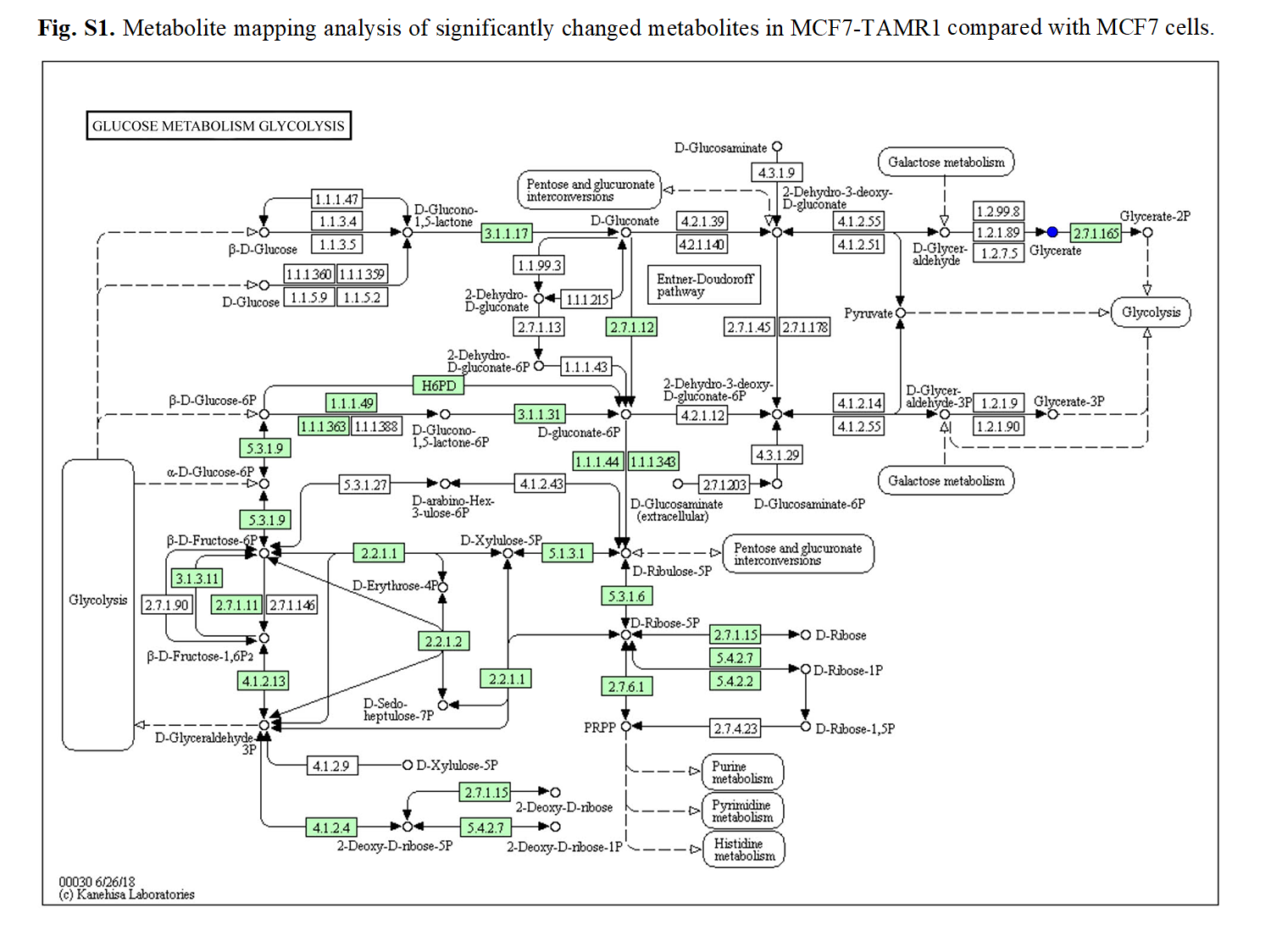

Supplement: Supplementary file 1 — Supplement Figure 1 [file 41419_2021_3781_MOESM1_ESM.tif]

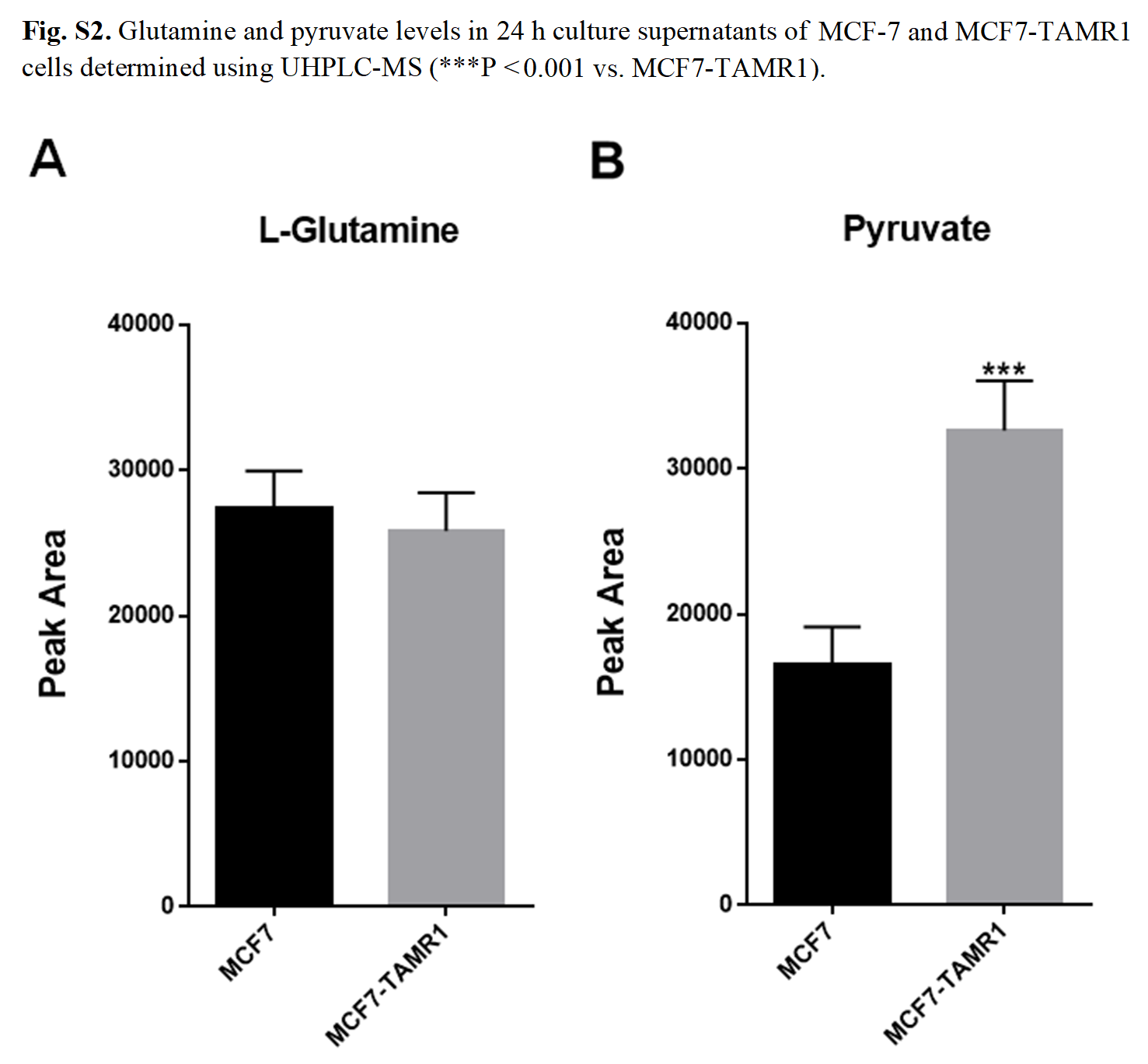

Supplement: Supplementary file 2 — Supplement Figure 2 [file 41419_2021_3781_MOESM2_ESM.tif]

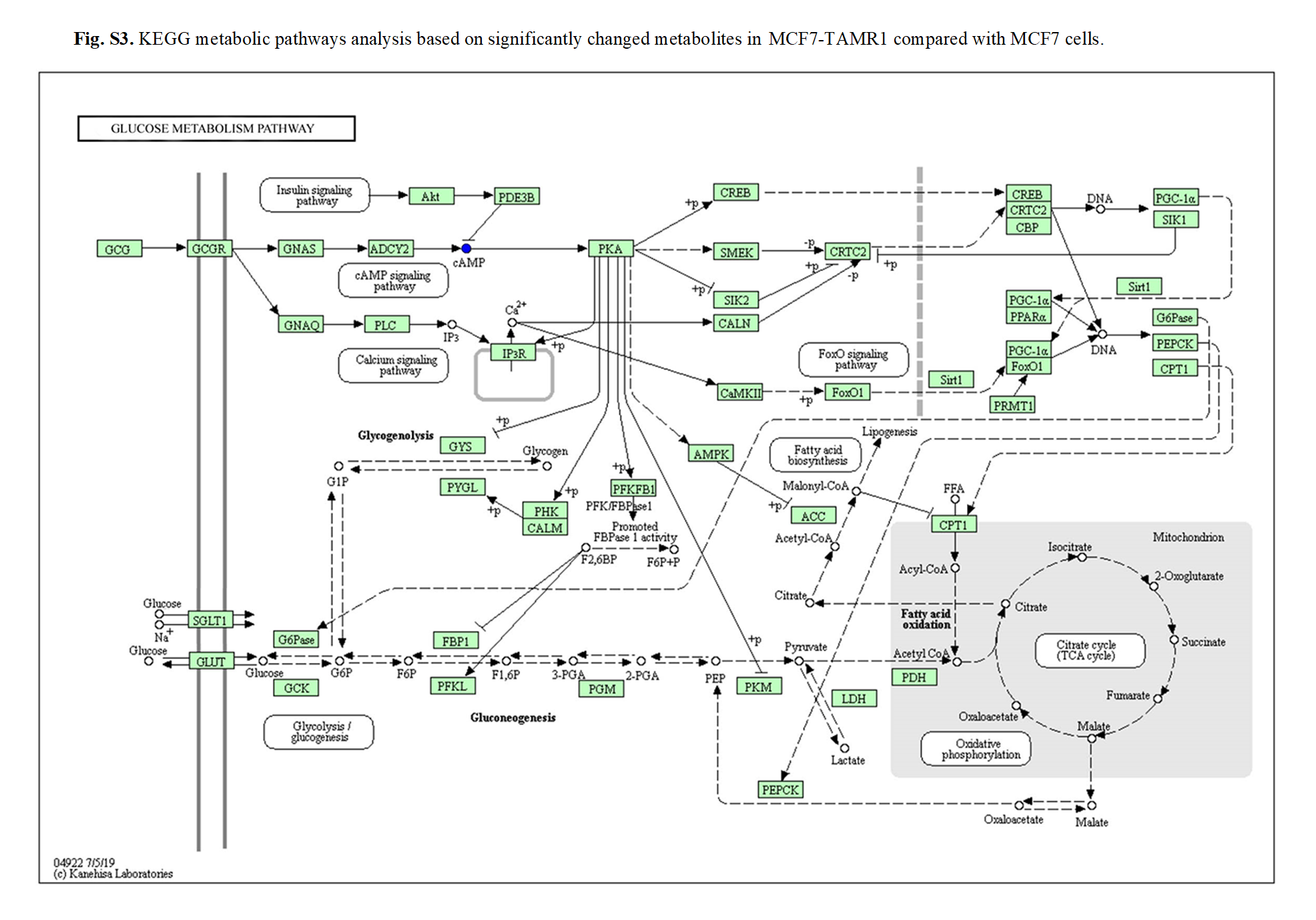

Supplement: Supplementary file 3 — Supplement Figure 3 [file 41419_2021_3781_MOESM3_ESM.tif]

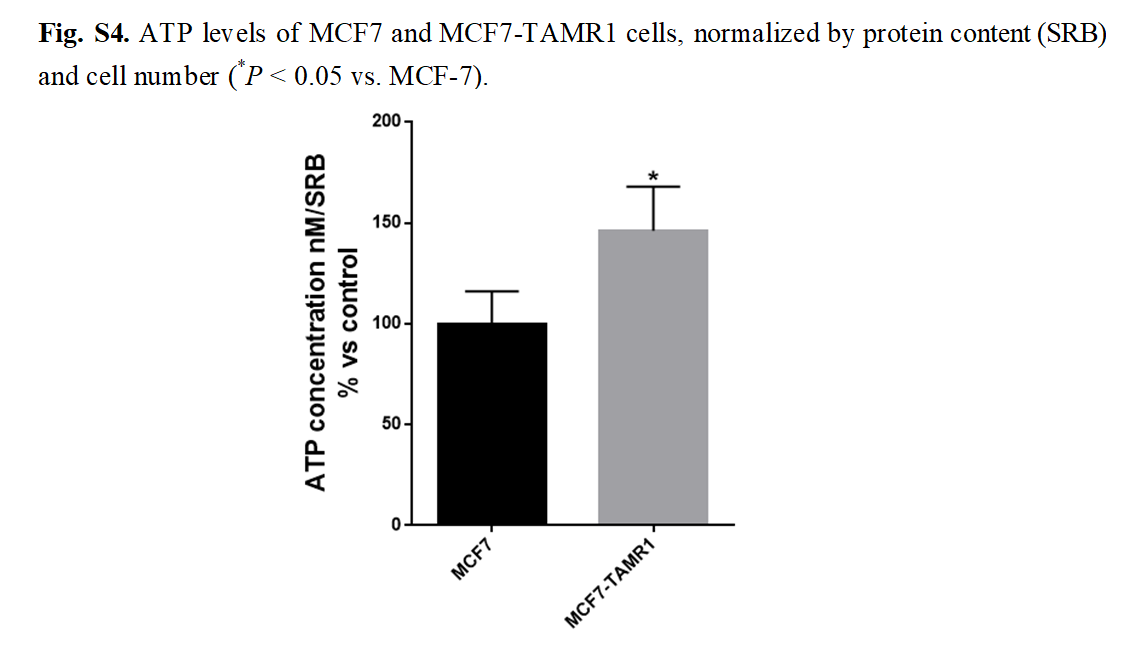

Supplement: Supplementary file 4 — Supplement Figure 4 [file 41419_2021_3781_MOESM4_ESM.tif]

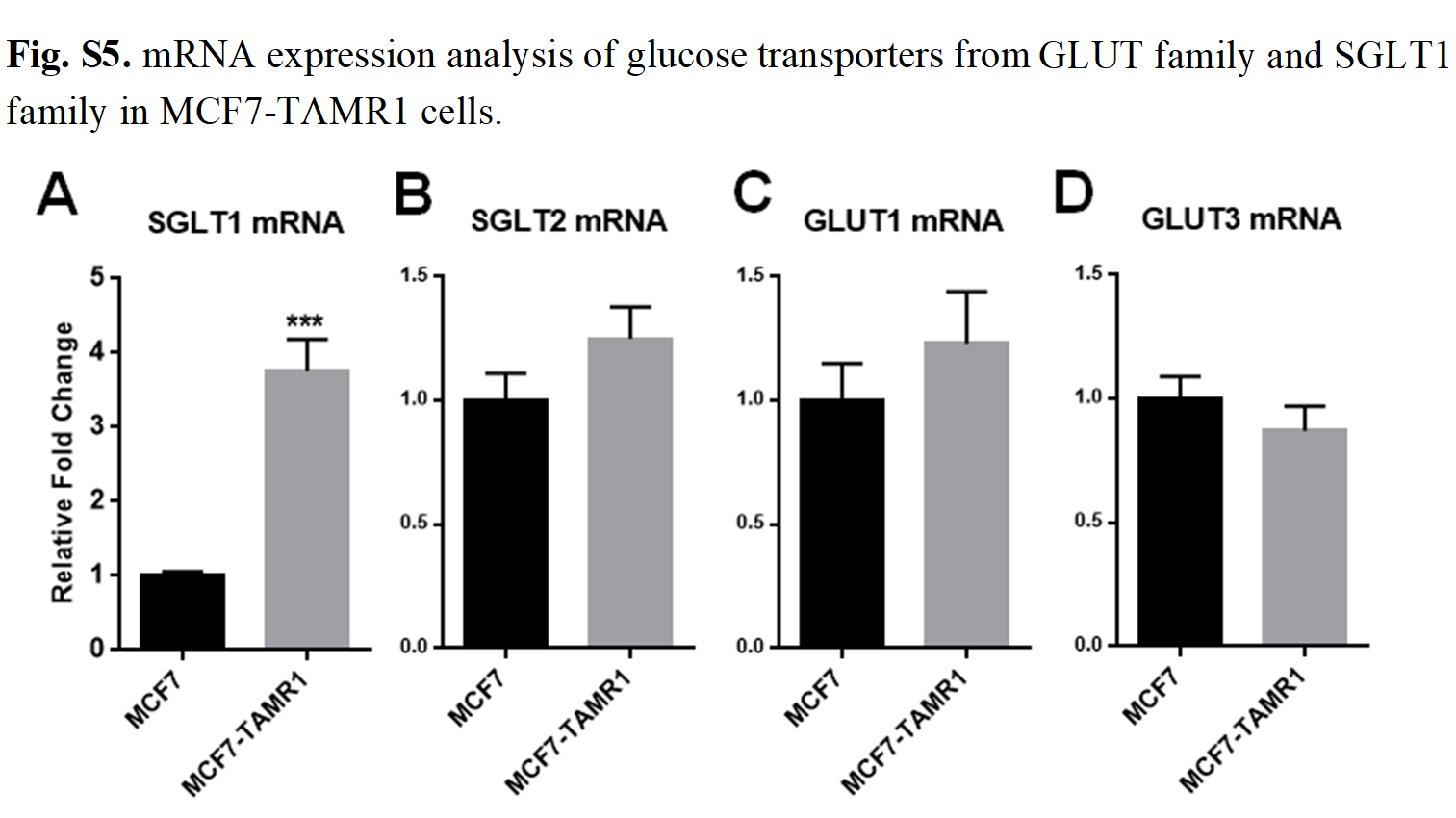

Supplement: Supplementary file 5 — Supplement Figure 5 [file 41419_2021_3781_MOESM5_ESM.tif]

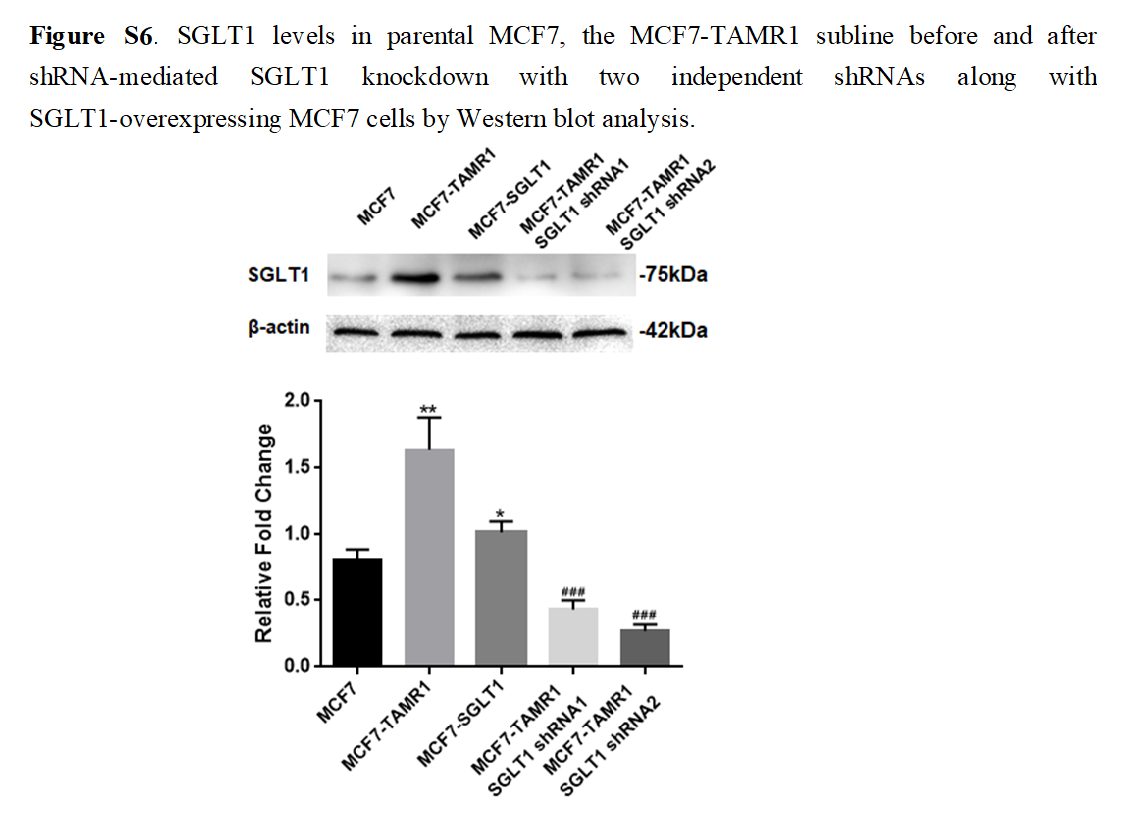

Supplement: Supplementary file 6 — Supplement Figure 6 [file 41419_2021_3781_MOESM6_ESM.tif]

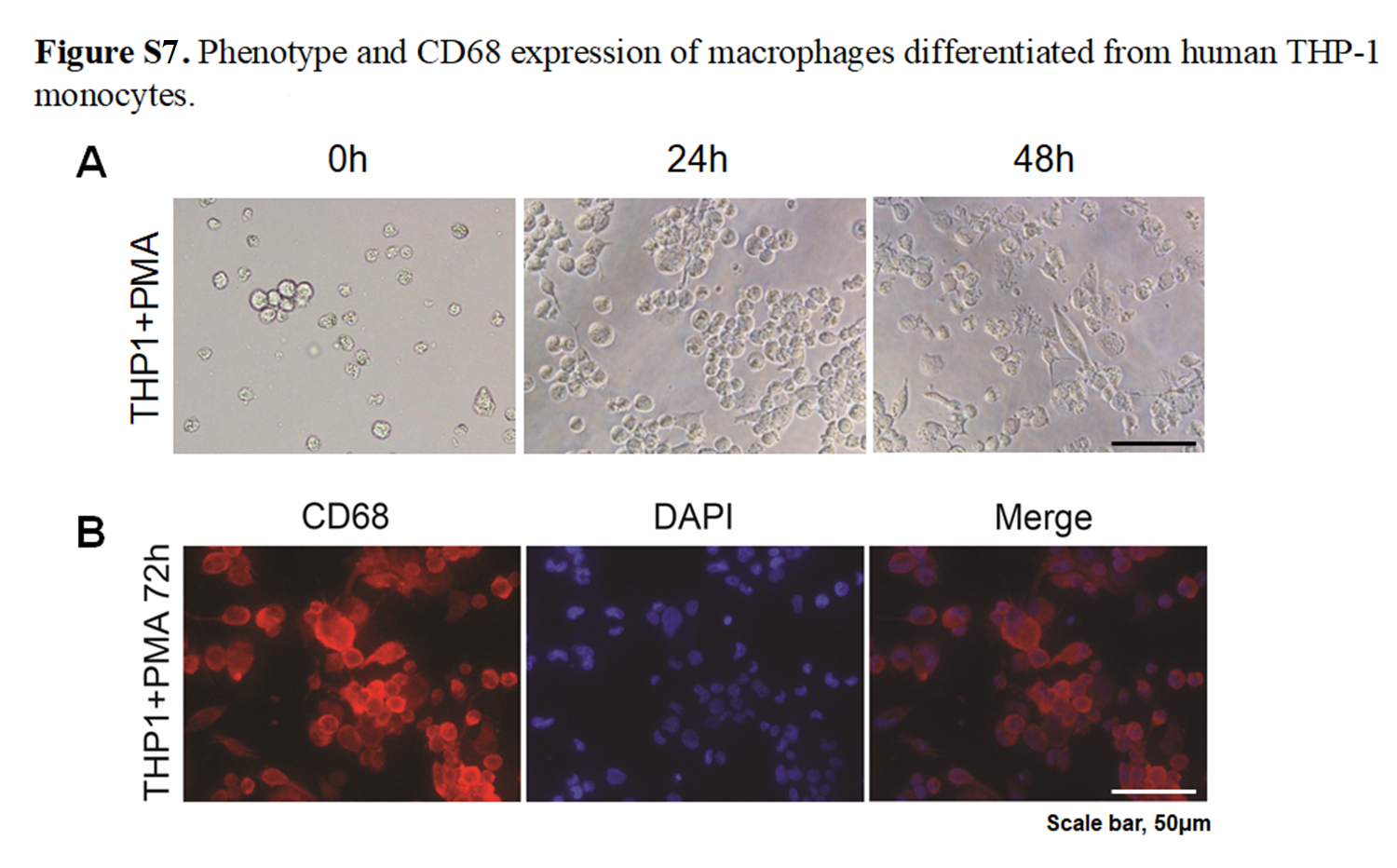

Supplement: Supplementary file 7 — Supplement Figure 7 [file 41419_2021_3781_MOESM7_ESM.tif]

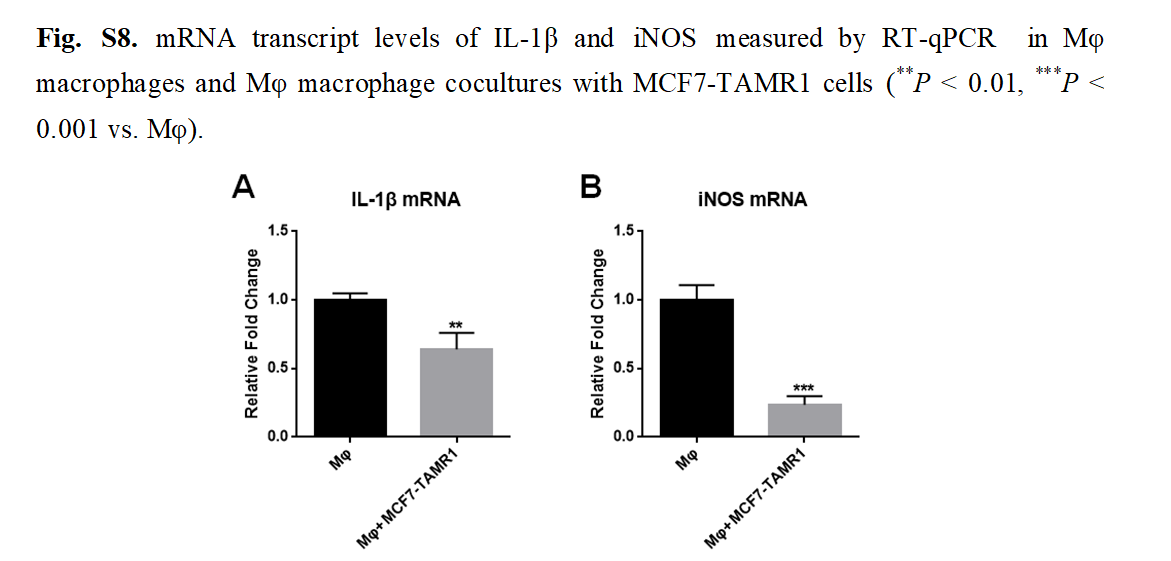

Supplement: Supplementary file 8 — Supplement Figure 8 [file 41419_2021_3781_MOESM8_ESM.tif]
