# Supplementary material for: Sodium/glucose cotransporter 1-dependent metabolic alterations induce tamoxifen resistance in breast cancer by promoting macrophage M2 polarization
Source: Cell Death Dis. 2021 May 18;12(6):509. doi: 10.1038/s41419-021-03781-x (PMC8131586; doi:10.1038/s41419-021-03781-x)
Supplement: Supplementary file 10 — Table S2 [file 41419_2021_3781_MOESM10_ESM.doc]

**Supplementary Table 2.** SGLT1 expression and CD163^+^ macrophage infiltration in 132 patients in Tamo-Re group and their association with clinicopathologic features

| Characteristics |  | SGLT1 expression | | |  | | CD163^+^ macrophage | |  |
| --- | --- | --- | --- | --- | --- | --- | --- | --- | --- |
|  | n(%) | High | Low | *P*-value | | High | | Low | *P*-value |
| Tumor size |  |  |  |  | |  | |  |  |
| ＜2cm | 28(21.2) | 10 | 18 | **0.032** | | 17 | | 11 | 0.656 |
| ≥ 2cm | 104(78.8) | 62 | 42 |  | | 69 | | 35 |  |
| Nodal status |  |  |  |  | |  | |  |  |
| N0, N1 | 70(53.0) | 26 | 44 | **<0.001** | | 40 | | 30 | **0.046** |
| N2, N3 | 62(47.0) | 46 | 16 |  | | 46 | | 16 |  |
| Histological grade |  |  |  |  | |  | |  |  |
| I | 22(16.7) | 11 | 11 | **<0.001** | | 16 | | 6 | 0.148 |
| II | 83(62.9) | 36 | 47 |  | | 49 | | 34 |  |
| III | 27(20.4) | 25 | 2 |  | | 21 | | 6 |  |
| ER status |  |  |  |  | |  | |  |  |
| ER+ | 104(78.8) | 55 | 49 | 0.525 | | 67 | | 37 | 0.825 |
| ER- | 28(21.2) | 17 | 11 |  | | 19 | | 9 |  |
| PR status |  |  |  |  | |  | |  |  |
| PR+ | 113(85.6) | 65 | 48 | 0.134 | | 70 | | 43 | 0.071 |
| PR- | 19(14.4) | 7 | 12 |  | | 16 | | 3 |  |
| HER2 status |  |  |  |  | |  | |  |  |
| HER2+ | 107(81.1) | 56 | 51 | 0.374 | | 65 | | 42 | **0.035** |
| HER2- | 25(8.9) | 16 | 9 |  | | 21 | | 4 |  |
| Ki67 |  |  |  |  | |  | |  |  |
| <20% | 64(48.5) | 32 | 32 | 0.382 | | 43 | | 21 | 0.716 |
| ≥20% | 68(51.5) | 40 | 28 |  | | 43 | | 25 |  |
